# Ebola: A Hyperinflated Emergency

**DOI:** 10.9745/GHSP-D-19-00422

**Published:** 2020-06-30

**Authors:** Victor K. Barbiero

**Affiliations:** a George Washington University Milken Institute of Public Health, Washington, DC, USA.

## Abstract

As with the Ebola outbreak, global under-5 mortality and morbidity should be considered a public health emergency of international concern.

## Key Messages

- Ebola virus disease outbreaks merit global concern, but worry and response can be hyperinflated.
- Ebola has killed about 15,266 people globally since 1976. Most recently, 2,267 people have died in the Democratic Republic of the Congo (DRC). However, these numbers pale in comparison to the under-5 deaths globally and in the DRC over the same period.
- Global child survival should be considered a public health emergency of international concern.
- Governments, donors, and multilateral organizations should significantly ramp up support for child survival interventions, including sustained system strengthening.

The 2019–2020 Ebola virus disease (EVD) outbreak in the Democratic Republic of the Congo (DRC) was a tragic and significant threat to thousands of people in the DRC and West Africa in general. As of March 25, 2020, an estimated 3,462 people in the DRC have been infected and an estimated 2,267 people have died from this terrible virus. Since EVD was first characterized in 1976, there have been 38 country-specific outbreaks, including the outbreak in the DRC. The total estimated EVD deaths from 1976 to 2020 is 15,266. The median number of deaths for all 38 outbreaks is 29 with a range of 0 to 4,809 (Table 1).^1^^,^^2^

**TABLE 1.** Country-Specific Ebola Virus Disease Outbreak Timeline

**Year**	**Country**	**Cases, No.**	**Deaths, No.**	**CFR, %**
1976	DRC	318	280	88.1
1976	Sudan	284	151	53.2
1977	DRC	1	1	100.0
1979	Sudan	34	22	64.7
1994	Gabon	52	31	59.6
1994	Ivory Coast	1	0	0.0
1995	DRC	315	254	80.6
1996	Gabon	31	21	67.7
1996	Gabon	60	45	75.0
1996	South Africa	1	1	100.0
2000	Uganda	425	224	52.7
2002	Gabon	65	53	81.5
2002	Congo	59	44	74.6
2003	Congo	143	128	89.5
2003	Congo	35	29	82.9
2004	Sudan	17	7	41.2
2005	Congo	12	10	83.3
2007	DRC	264	187	70.8
2007	Uganda	149	37	24.8
2008	DRC	32	14	43.8
2011	Uganda	1	1	100.0
2012	Uganda	24	17	70.8
2012	Uganda	7	4	57.1
2012	DRC	57	29	50.9
2014	Nigeria	20	8	40.0
2014	Mali	8	6	75.0
2014	Senegal	1	0	0.0
2014	USA	4	1	25.0
2014	UK	1	0	0.0
2014	DRC	69	49	71.0
2014	Spain	1	0	0.0
2015	Italy	1	0	0.0
2014–2016	Guinea	3,811	2,543	66.7
2014–2016	Liberia	10,675	4,809	45.0
2014–2016	Sierra Leone	14,124	3,956	28.0
2017	DRC	8	4	50.0
2018	DRC	54	33	61.1
2018–2020^a^	DRC	3,462	2,267	65.5
Total 1976–2020		34,626	15,266	44.1

Abbreviations: CFR, case fatality rate; DRC, the Democratic Republic of the Congo; UK, United Kingdom.

a Until March 4, 2020.

The EVD case fatality rate (CFR) can be 0 or reach 100%, depending on the scope and location of the outbreak (e.g., 2011 Uganda [1 case 1 death], Senegal 2014 [1 case, 0 deaths]). Although there are 4 different types of Ebola virus,^3^ generally speaking, the EVD CFR averages about 50%.^4^ The 2015 outbreak in Guinea, Liberia, and Sierra Leone infected an estimated 28,610 people and killed 11,308 with a CFR of approximately 40%. Without question, EVD is an important and daunting public health issue for Africa and potentially for the world.

However, EVD is an epizootic infection with periodic human exposure and transmission. Since its emergence in 1976; the virus remains an uncomfortable human pathogen. It kills too fast, kills too many, and is not easily transmitted; thus, human outbreaks are limited, and its pandemic potential is moderate to low. It has not yet achieved equilibrium with its human host as it apparently has with its bat host. Furthermore, EVD’s 40%–50% CFR in humans may be considered evolutionarily unsound in many respects for a successful human pathogen.

But, the real issue concerning EVD is disease sensationalism. This can be characterized as an unfounded perception of a global emergency, not necessarily anchored in the epidemiology, pandemic potential, and total mortality of a pathogen. Rather, it appears the classification of a global emergency is based more on the political ramifications, the newsworthiness of the disease *de jour*, and yes, financial aspects and funding streams of a declared emergency for an emerging and/or reemerging infection. Tragically, more than 11,000 died of EVD in Guinea, Liberia, and Sierra Leone. However, it should be recognized that far more individuals (especially children under 5 years old) died since 1976 in these countries from preventable and treatable but less exotic infections. Should there not be a “moral claim” by the world’s children on emergency resources as well?

In 2014, the Obama administration submitted an emergency funding request, and in 2015, Congress authorized an appropriation of approximately US$5.4 billion in an omnibus emergency bill to combat EVD spread, protect America from an EVD outbreak, and support the development of an EVD vaccine.^5^ Notably, this appropriation exceeded the total 2015 authorization of US$3.13 billion for all U.S. government assistance for maternal, child, reproductive health, malaria, nutrition, and neglected tropical diseases by US$227 million.^6^ Did EVD epidemiology and national/global risk justify the emergency bill investment? Perhaps, perhaps not.

Clearly emerging and reemerging infections are important and need to be handled with interventions that mitigate spread and minimize mortality, coupled with adequate and sustained epidemic preparedness. Indeed, there have been concrete benefits for managing future internationally-important outbreaks such as better infection prevention techniques and equipment, improved surveillance methods, better international response, and a better understanding of behavioral determinants and the basic biology of such viruses and techniques for vaccine and therapeutic development. Furthermore, in some countries, lessons learned from the severe acute respiratory syndrome and Middle East respiratory syndrome outbreaks are being applied to the present COVID-19 pandemic.

Hyperinflated, news-based fear; questionable statistical models; and global emergency statements should not justify disproportionate allocations of time and effort on a specific issue of lesser epidemiological impact when millions of people are at risk from diseases that can be prevented, treated, and cured.

It is a sad and tragic fact that from 1976 to present, approximately 34,600 individuals have been infected with EVD and approximately 15,200 have died from EVD (CFR=44.1%) (Table 1). It is noteworthy that the U.S. Centers for Disease Control and Prevention predicted that the 2014–2016 West African EVD outbreak could have infected more than 1.4 million people in Liberia and Sierra Leone alone^7^:

*Extrapolating trends to January 20, 2015, without additional interventions or changes in community behavior (e.g., notable reductions in unsafe burial practices), the model also estimates that Liberia and Sierra Leone will have approximately 550,000 Ebola cases (1.4 million when corrected for underreporting).*

Over the period from 1976 to 2017, in the DRC alone approximately 12.43 million children under 5 years old have died, mostly from preventable and curable childhood diseases (Table 2)^8^ (Figure).^2^ This number dwarfs the 15,266 people who have died from EVD globally over the same period. Furthermore, at the global level, annually, an estimated 5.3 million children under 5 years old die from preventable and curable causes worldwide.^9^ Which qualifies as a more urgent and important global health emergency: global EVD or global under-5 mortality?

**TABLE 2.** Under-5 Deaths in the Democratic Republic of the Congo from 1976–2017^8^^a^

	**Year**											
**Estimated** **Level**	**1976**	**1977**	**1978**	**1979**	**1980**	**1981**	**1982**	**1983**	**1984**	**1985**	**1986**	**Total Deaths**
**Low**	196,957	200,518	204,337	208,117	211,628	215,118	219,168	222,591	226,628	231,114	235,707	**2,371,883**
**Medium**	233,087	235,767	238,287	241,006	243,644	246,468	249,515	252,490	255,711	259,276	263,126	**2,718,377**
**High**	275,619	276,160	277,290	278,019	279,506	280,937	282,416	284,257	286,999	289,612	292,898	**3,103,713**
** **												** **
	**1987**	**1988**	**1989**	**1990**	**1991**	**1992**	**1993**	**1994**	**1995**	**1996**	**1997**	**Total Deaths**
**Low**	240,269	245,357	250,850	256,597	262,380	268,588	275,015	281,307	287,517	292,755	296,860	**2,957,495**
**Medium**	267,689	272,359	277,624	283,090	289,076	295,539	302,429	309,281	315,772	321,800	326,733	**3,261,392**
**High**	296,555	301,225	306,764	312,320	318,881	326,150	333,311	341,237	348,421	355,187	360,844	**3,600,895**
** **												** **
	**1998**	**1999**	**2000**	**2001**	**2002**	**2003**	**2004**	**2005**	**2006**	**2007**	**2008**	**Total Deaths**
**Low**	299,906	301,930	302,590	302,362	301,596	300,522	298,587	295,532	291,987	287,351	281,512	**3,263,875**
**Medium**	330,528	332,688	333,792	333,850	333,311	332,597	331,277	329,811	328,038	326,465	324,650	**3,637,007**
**High**	365,124	368,169	369,844	370,391	370,239	369,727	368,988	368,562	369,274	371,071	373,064	**4,064,453**
** **												** **
	**2009**	**2010**	**2011**	**2012**	**2013**	**2014**	**2015**	**2016**	**2017**			**Total Deaths**
**Low**	274,783	267,800	259,746	251,199	243,411	234,782	225,454	216,143	206,535			**2,179,853**
**Medium**	322,801	320,391	318,265	315,758	313,762	310,711	307,687	303,618	300,265			**2,813,258**
**High**	375,987	380,011	383,529	387,635	393,306	399,404	405,716	415,115	422,796			**3,563,499**
** **												** **
**Totals 1976–2017**												**Total Deaths**
**Low**												**10,773,106**
**Medium**												**12,430,034**
**High**												**14,332,560**

a As of December 19, 2019.

**FIGURE.**
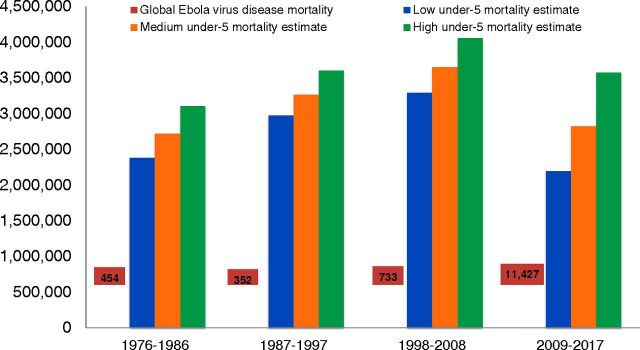
Estimated Cumulative Under-5 Mortality in the Democratic Republic of the Congo Versus Global Ebola Virus Disease Mortality, 1976–2017^a^^,^^2^^,^^8^ ^a^Note: Ebola virus disease mortality not to scale.

The term public health emergency of international concern (PHEIC) is defined in the International Health Regulations (2005) as:

An extraordinary event, which is determined, as provided in these Regulations: to constitute a public health risk to other States through the international spread of disease; and to potentially require a coordinated international response. This definition implies a situation that: is serious, unusual or unexpected; carries implications for public health beyond the affected State’s national border; and may require immediate international action.^10^

The PHEIC definition accurately describes an epidemiological emergency. However, it falls short on quantifying the impact of the emergency on existing and/or potential morbidity and mortality on a national, regional, or global scale. As noted, the burden of disease for children under 5 years old dramatically exceeds the cumulative global morbidity and mortality from EVD. Considering measles alone, from January 2019 through November 2019, the United Nations Children’s Fund reported 5,000 measles deaths (90% in children under 5 years old) in the DRC, with over 200,000 measles cases.^11^^,^^12^ Globally, measles surged in 2019 and killed about 140,000 worldwide,^13^ which is about 9.2 times the total number of deaths caused by EVD in its 43-year history as a human pathogen.

Clearly, great success has been achieved over the last 25 years in reducing deaths in children under 5 years old. However, in my view, the “unfinished agenda for child survival,”^14^ also qualifies as a public health emergency that should be of international concern. It deserves heightened attention by the governments, multilateral and bilateral donors alike, and should not be marginalized. Every day, approximately 14,500 children under 5 years old die, the equivalent of 35 Boeing 747 plane crashes.^15^ Clearly, global child mortality fits the World Health Organization’s definition of a “Grade 3” emergency and should be categorized as such.^16^ We need increased and continuous global investment in child survival and sustainable health system development.^17^ The moral claim of the world’s children should no longer be ignored.

The “unfinished agenda for child survival,” also qualifies as a public health emergency that should be of international concern.

## Notes

**First published online:** May 19, 2020
